# Integrative transcriptomic analysis identifies shared coding and non-coding biomarkers in tongue squamous cell carcinoma across diverse populations

**DOI:** 10.1007/s12672-026-05428-9

**Published:** 2026-08-27

**Authors:** Ronit Roy, Monika Rajput, Manoj Pandey

**Affiliations:** 1 DHR-ICMR Advanced Molecular Oncology Diagnostic Services (DIAMOnDS), Institute of Medical Sciences, Banaras Hindu University, Varanasi, 221005 India; 2 Department of Surgical Oncology, Institute of Medical Sciences, Banaras Hindu University, Varanasi, 221005 India; 3 Devi Ahilya Vishwavidyalaya, Takshashila Campus, Khandwa Road, Indore, M.P. 452017 India

**Keywords:** Head and neck cancer, Differentially expressed genes, Functional enrichment analysis, Transcriptomic profiling, Bioinformatics analysis, Biomarkers

## Abstract

**Background:**

Tongue cancer, a prevalent subtype of oral malignancy, remains a significant global health burden with high morbidity and mortality, particularly in developing countries. Understanding its molecular landscape is crucial for developing targeted diagnostics and therapeutics.

**Objective:**

This systematic review aimed to identify common differentially expressed protein-coding genes (DEGs) in tongue cancer across multiple populations and explore their functional significance through integrative bioinformatic analyses.

**Methods:**

We performed an integrative meta-analysis of transcriptomic datasets from China, the USA, and Australia to identify common differentially expressed genes (DEGs). Functional enrichment, protein–protein interaction (PPI), and regulatory network analyses were conducted. Crucially, the clinical relevance of identified hub genes was validated using the TCGA-HNSC cohort to assess correlations with tumor stage and overall survival.

**Results:**

A total of 133 common DEGs were identified, primarily enriched in extracellular matrix (ECM) disassembly, collagen catabolism, and IL-17 signaling. Key hub genes included *MMP3*, *MMP10*, *COL1A1*, *CDKN2A*, and *CXCL11*. Network analysis uncovered novel lncRNAs, including *TENM3-AS1* and *DUXAP10*, regulating immune and epithelial pathways. Clinical validation demonstrated that high expression of *STC2* and *MMP3* significantly correlated with advanced tumor stage (*p* < 0.05). Furthermore, *STC2* upregulation was identified as a significant predictor of poor overall survival (*p* = 0.0037).

**Conclusion:**

This study defines a cross-population gene signature driving TSCC through ECM remodeling and immune modulation. The validation of *STC2* as a prognostic marker highlights its potential for clinical risk stratification.

**Graphical Abstract:**

**Figure:**
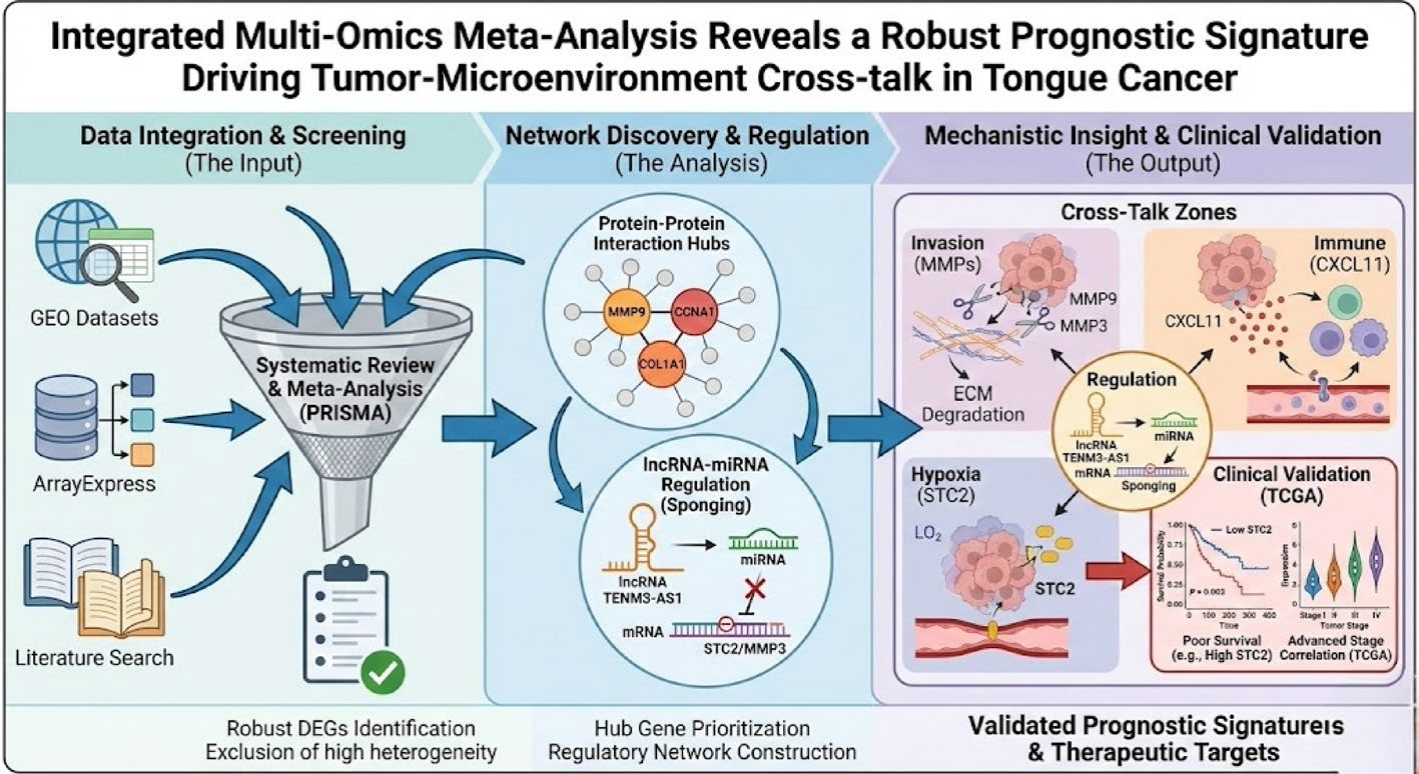

**Supplementary Information:**

The online version contains supplementary material available at https://doi.org/10.1007/s12672-026-05428-9.

## Introduction

Tongue cancer, a major global health concern, ranks among the most common oral cavity malignancies, with an estimated 389,846 new cases and 188,438 deaths worldwide in 2022 [1]. Despite advances in early detection and treatment, tongue cancer is associated with high morbidity and mortality, particularly in developing countries with limited healthcare access and awareness. Recent therapeutic advances, including targeted therapies and immunotherapy alongside conventional surgical and chemoradiation approaches, have improved outcomes in select patient populations. However, tongue cancer is a multifactorial disease characterized by complex molecular and cellular mechanisms, necessitating further research to elucidate its pathogenesis and develop effective strategies for early detection, prognosis, and personalized therapy. Multiple molecular mechanisms contribute to tongue cancer development and progression, including genomic alterations [2], dysregulation of mRNA and non-coding RNA expression [3, 4], and epigenetic modifications [5, 6]. Key signaling pathways, such as PI3K-Akt, MAPK, Wnt, Notch, and immune response pathways, regulate cell proliferation, differentiation, invasion, and metastasis in tongue cancer [7–9]. Identifying differentially expressed genes (DEGs) and their associated pathways is essential for advancing our understanding of tongue cancer pathogenesis. Numerous gene expression profiling studies have identified hundreds of DEGs and potential biomarkers, including MMP10 [10], ITGA3 [11], S100A7 [12], miR-21 [13], and p16INK4A [14]. However, these studies often focus on single cohorts from specific populations with limited sample sizes and varying methodologies, potentially introducing bias and limiting generalizability. Therefore, comprehensive integrative analyses using advanced bioinformatic approaches are critical to minimize bias and enhance understanding of tongue cancer’s molecular landscape. By leveraging *in silico* tools and functional enrichment analysis, this study systematically identifies key gene expression patterns, biological functions, molecular interactions, and critical pathways in tongue cancer tissues across diverse populations. These efforts are essential for discovering reliable biomarkers and developing improved diagnostic, prognostic, and therapeutic strategies.

##  Materials and methods

###  Search strategy

An extensive search was conducted in the Gene Expression Omnibus (GEO) (https://www.ncbi.nlm.nih.gov/geo/) database using Medical Subject Heading (MeSH) terms, including “Tongue Neoplasm,” “Tongue Cancer,” and “Tongue Carcinoma.” Boolean operators (“OR”) was applied to construct the following search query: “Tongue Neoplasm” OR “Tongue Carcinoma” Or “Tongue Cancer.” This search aimed to retrieve transcriptomic datasets focused on differentially expressed genes (DEGs) in tongue cancer tissues. This study follows the PRISMA (Preferred Reporting Items for Systematic Reviews and Meta-Analyses) guidelines.

###  Inclusion criteria

Studies were included if they met all the following criteria:

- Human case-control studies utilizing transcriptomic profiling (RNA-seq) of tongue cancer tissue vs. normal tongue tissue.
- A clearly defined list of differentially expressed genes (DEGs), including adjusted False discovery rate (FDR < 0.05) and log2 fold change (log2FC) values (+ 3 to −3).
- Use of bulk RNA data to ensure compatibility and reduce heterogeneity across datasets.
- Availability of raw or processed data in GEO for further analysis.

### Exclusion criteria

A total of 32 studies were initially retrieved. After title, abstract, screening 10 studies were selected for full-text screening, and only 5 studies were retained based on the following exclusion criteria:

- In vitro, in vivo, or *in silico* studies without human tissue-based transcriptomic profiling (*n* = 18).
- Studies using single-cell RNA sequencing (scRNA-seq), due to its inherent biological and technical variability, limited compatibility with bulk RNA-seq data, and distinct normalization requirements (*n* = 5).
- Datasets without statistically significant DEGs or lacking required log2FC and FDR values (*n* = 3).
- Incomplete datasets, inaccessible raw data, or lack of metadata (e.g., sample type, patient demographics) (*n* = 5).
- Studies including tumor sites beyond the tongue, such as oropharyngeal carcinomas (*n* = 1).

###  Screening of articles for eligibility

A two-phase screening process was employed:

1. Phase I – Title and Abstract Review: Articles were screened for relevance to tongue cancer and the presence of transcriptomic analysis.
2. Phase II – Full-Text Review: Studies were excluded if they were systematic reviews, meta-analyses, or focused solely on experimental models not aligned with the defined inclusion criteria.
3. All screening steps were performed independently by two researchers, with discrepancies resolved by consensus.

###  Data extraction

The datasets were generated on different Illumina sequencers (HiSeq 2500 vs. NovaSeq 6000) and included circRNA-seq libraries. These platforms differ in chemistry and throughput: NovaSeq uses 2-channel SBS (vs. HiSeq’s 4-color chemistry), which can lead to “poly-G” errors in low-quality reads, and NovaSeq yields far more data per run (up to ~ 6 Tb, ~ 2 × 10^10 reads compared to ~ 300 Gb for a HiSeq run). Additionally, circRNA-seq libraries involve RNase R treatment to degrade linear RNAs, enriching circular transcripts. This RNase R step inherently biases the library composition (removing many linear RNAs and affecting some circRNAs), so we treated circRNA results separately and noted this as a platform difference. Each GEO dataset was processed within itself using GEO2R’s defaults: for RNA-seq, this means DESeq2 with TPM normalization, and for microarrays, limma on the submitted expression values. In practice, we compared tumor vs. control within each series. We did not merge raw data across studies or apply global batch-correction (e.g., ComBat) to the combined datasets. In other words, we relied on per-dataset normalization (log2 TPM or quantile-normalized intensity) and then compared results across studies. We acknowledge this as a limitation, since without cross-study harmonization, residual platform- or batch-related biases could remain. We deliberately omitted scRNA-seq datasets because single-cell profiles are not directly comparable to bulk RNA-seq. Single-cell RNA-seq captures individual cell transcriptomes with high sparsity (many zero-count “dropouts” for low-abundance genes), while bulk RNA-seq measures the average over all cells in a sample. These technical and biological differences mean that mixing scRNA-seq with bulk data would confound the analysis. Since our goal was to compare tissue-level transcriptomes across populations, only bulk (or bulk-like circRNA) datasets were included.

Although GSE131568 (https://www.ncbi.nlm.nih.gov/geo/query/acc.cgi?acc=GSE131568) and GSE156178 (https://www.ncbi.nlm.nih.gov/geo/query/acc.cgi?acc=GSE156178) were originally annotated as circRNA and exosomal RNA datasets, respectively, both include tissue derived samples from tongue squamous cell carcinoma (TSCC) patients. Therefore, only the tongue specific bulk transcriptome data from these studies were extracted and analyzed. This approach ensured inclusion of biologically relevant TSCC expression profiles while maintaining the defined focus on human tongue tumor versus normal tissue. Platform specific biases were minimized by applying per dataset normalization prior to comparative analysis. In addition to these datasets, three independent GEO cohorts were incorporated to capture a broader representation of TSCC patient characteristics.

The GSE143950 (https://www.ncbi.nlm.nih.gov/geo/query/acc.cgi?acc=GSE143950) dataset (China) comprised 8 samples (4 tumor and 4 normal) from patients aged 65–70 years, with a sex distribution of 3 males and 1 female, and tumors located in the anterior tongue, profiled using Illumina HiSeq 2500 sequencing.

The GSE184616 (https://www.ncbi.nlm.nih.gov/geo/query/acc.cgi?acc=GSE184616) dataset (Australia) included 30 samples (13 tumor and 13 normal) generated on the Illumina NovaSeq 6000 platform, representing patients above and below 50 years of age (6 males and 5 females), also with anterior tongue tumors.

The GSE149008 (https://www.ncbi.nlm.nih.gov/geo/query/acc.cgi?acc=GSE149008) dataset (China) contributed 12 samples (6 tumor and 6 normal), sequenced using the Illumina HiSeq 4000 system, from patients aged 40–65 years, including 5 males and 1 female, with anterior tongue lesions. Collectively, these datasets provide a diverse and well characterized TSCC patient population, allowing robust identification of differentially expressed genes while accounting for demographic and technical variability.

### Methodological quality and risk of bias assessment

To evaluate the robustness and internal validity of the included public transcriptomic datasets before cross-population integration, a modified version of the Newcastle-Ottawa Scale (NOS) customized for high-throughput genomic studies was implemented. Two independent investigators scored each dataset across three essential domains:

(1) Cohort Selection (Max 3 stars): Verification of tissue diagnosis (TSCC vs. healthy adjacent tissue) and clear documentation of clinical staging. (2) Subsite Comparability (Max 2 stars): Controlling for anatomical heterogeneity by restricting samples specifically to the anterior tongue. (3) Expression Profiles and Technical Quality (Max 4 stars): Use of verified sequencing platforms (Illumina HiSeq/NovaSeq), adequate sequencing depth, raw or clean metadata availability, and rigorous False Discovery Rate (FDR < 0.05) filtering. Datasets scoring ≥ 7 stars overall were categorized as high-quality with low risk of baseline technical bias, justifying their inclusion in the overlapping Venn analysis despite differences in chemistry.

###  Venn diagram analysis

A Venn diagram was constructed using Python [15] to identify common DEGs across three populations (China, USA, Australia). Venn analysis was performed to detect shared DEGs among all three populations and pairwise comparisons (China & USA, China & Australia, USA & Australia). For integration with bulk mRNA datasets, differentially expressed circRNAs were mapped to their corresponding host gene symbols, and overlapping analyses were performed at the host gene level to enable consolidated Venn comparison.

###  Functional enrichment analysis

Functional enrichment was conducted using Enrichr [16] (https://maayanlab.cloud/Enrichr/) to explore the biological significance of common protein-coding genes in tongue cancer. Gene Ontology (GO) analysis categorized genes into Biological Process (BP), Molecular Function (MF), and Cellular Component (CC). Pathway enrichment analysis was performed using the KEGG (https://www.genome.jp/kegg/pathway.html) database [17], and Disease Ontology (DO) analysis identified disease associations. Results were visualized to highlight shared biological processes, molecular functions, cellular components, pathways, and disease relevance. 2.8 Network Construction, Protein-Protein Interaction (PPI), and Gene–miRNA Interaction. Common coding genes were analyzed using NetworkAnalyst [18] (https://www.networkanalyst.ca/) to construct interaction networks. A Protein–Protein Interaction (PPI) network identified potential hub genes, and a gene–miRNA interaction network explored regulatory relationships between genes and microRNAs.

### Network construction, PPI, and gene–miRNA interaction network

Common protein coding genes were analyzed using NetworkAnalyst [18] (https://www.networkanalyst.ca/) to construct interaction networks. A Protein-Protein Interaction (PPI) network identified potential hub genes, and a gene–miRNA interaction network explored regulatory relationships between genes and microRNAs. Protein-protein interaction and gene miRNA interaction networks that were constructed using the NetworkAnalyst platform with the STRING interactome as the reference database and default parameters. Hub genes were identified using the built-in network topology ranking functions of NetworkAnalyst. Nodes with the highest connectivity scores, based on degree centrality, were prioritized as hub genes, as these represent highly connected components within the interaction network and may play key regulatory roles in tongue cancer related pathways.

##  Results

**Fig. 1:**
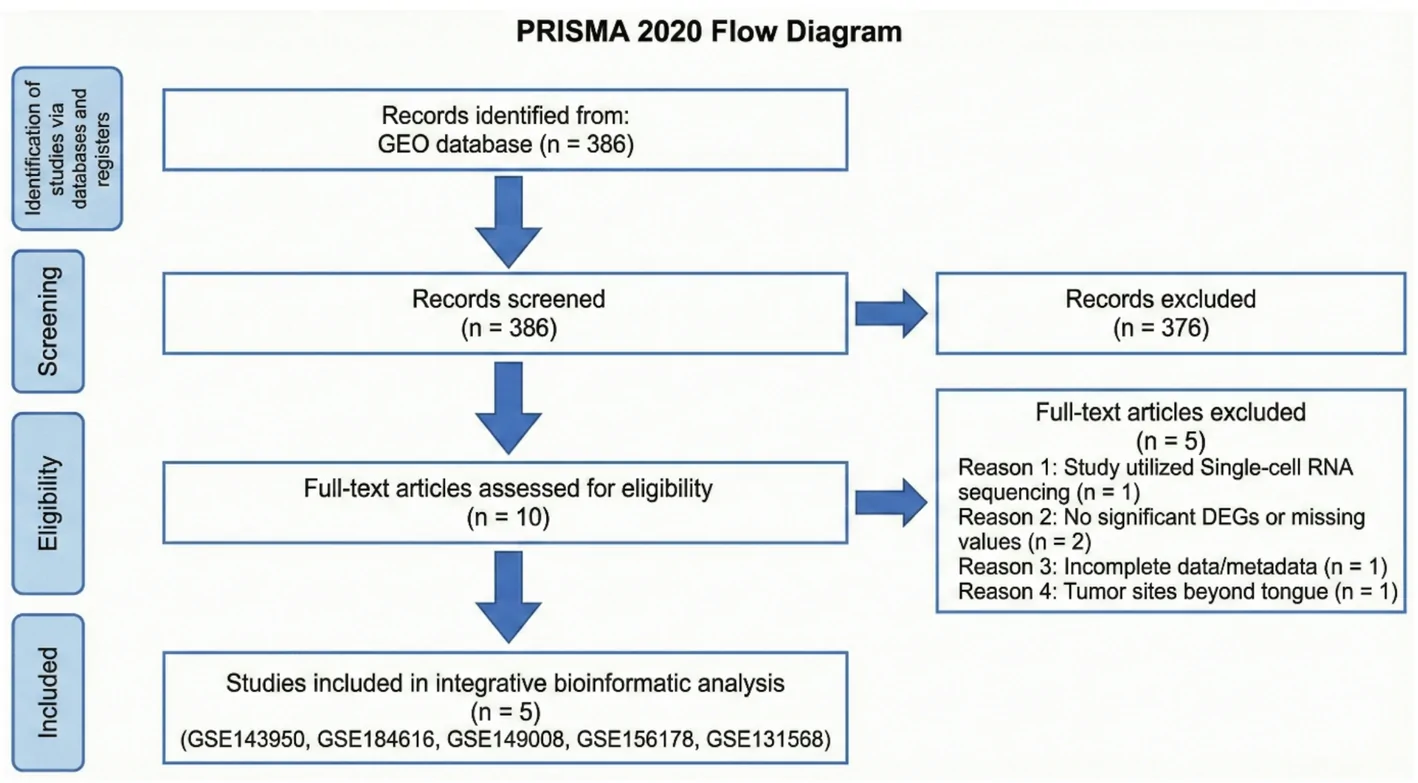
PRISMA 2020 flow chart showing the selection of datasets from the GEO database. The five datasets (GSE143950, GSE184616, GSE149008, GSE156178, and GSE131568) were retained for the final analysis

From 386 records retrieved from the GEO database, 376 were excluded after screening titles and abstracts. Following full-text review, 5 transcriptomic studies meeting eligibility criteria were included (Fig. 1; Table 1).

**Table 1:** Baseline characteristics of patients selected for the study, grouped by population

Accession No.	Country	Samples	Technique	Age	Sex	Tumour site
GSE143950	China	8 samples (4 cancer, 4 normal)	RNA seq. (Illumina HiSeq 2500 sequencing)	65–70	3 Male, 1 Female	Anterior
GSE184616	Australia	30 samples (13 normal, 13 tumor)	RNA seq. (Illumina NovaSeq 6000 platform)	> 50-<50	6 Male, 5 Female	Anterior
GSE149008	China	12 samples (6 tumor, 6 normal)	RNA seq. (Illumina HiSeq 4,000 system)	40–65	5 Male, 1 Female	Anterior
GSE156178	USA	24 samples (19 cancer, 5 normal)	RNA seq. (Illumina 2500 sequencing)	> 65-<65	37 Male, 35 Female	Anterior
GSE131568	China	12 samples (6 tumor, 6 normal)	circRNA seq. (Illumina HiSeq 4000 sequencer)	40–70	2 Male, 4 Female	Anterior

###  DEGs detection in tongue cancer

Differentially expressed genes (DEGs) were identified from transcriptomic datasets across three populations: China (*n* = 2,212) [19–21], USA (*n* = 701) [22], and Australia (*n* = 463) [23]. Only genes with a FDR value < 0.05 and a log2 fold change (log2FC) between + 3 and − 3 were considered. A stringent fold change threshold was applied to focus on genes showing strong and consistent expression differences across datasets generated from different populations and sequencing platforms. This approach was chosen to reduce the inclusion of false positive findings that may arise from technical variation between studies. By prioritizing genes with larger and more consistent changes in expression, we aimed to identify robust molecular signals that are more likely to reflect stable biological differences associated with tongue cancer.

Applying the modified NOS framework confirmed that all five included GEO series (GSE143950, GSE184616, GSE149008, GSE156178, and GSE131568) exhibited high methodological quality. Every selected dataset earned the maximum score for subsite uniformity by focusing exclusively on the anterior tongue, effectively neutralizing anatomical confounding factor variables. Minor variance was limited to platform throughput differences (HiSeq vs. NovaSeq), which were successfully resolved by applying independent, within-dataset normalization protocols prior to signature merging.

###  Identification of common DEGs

**Fig. 2:**
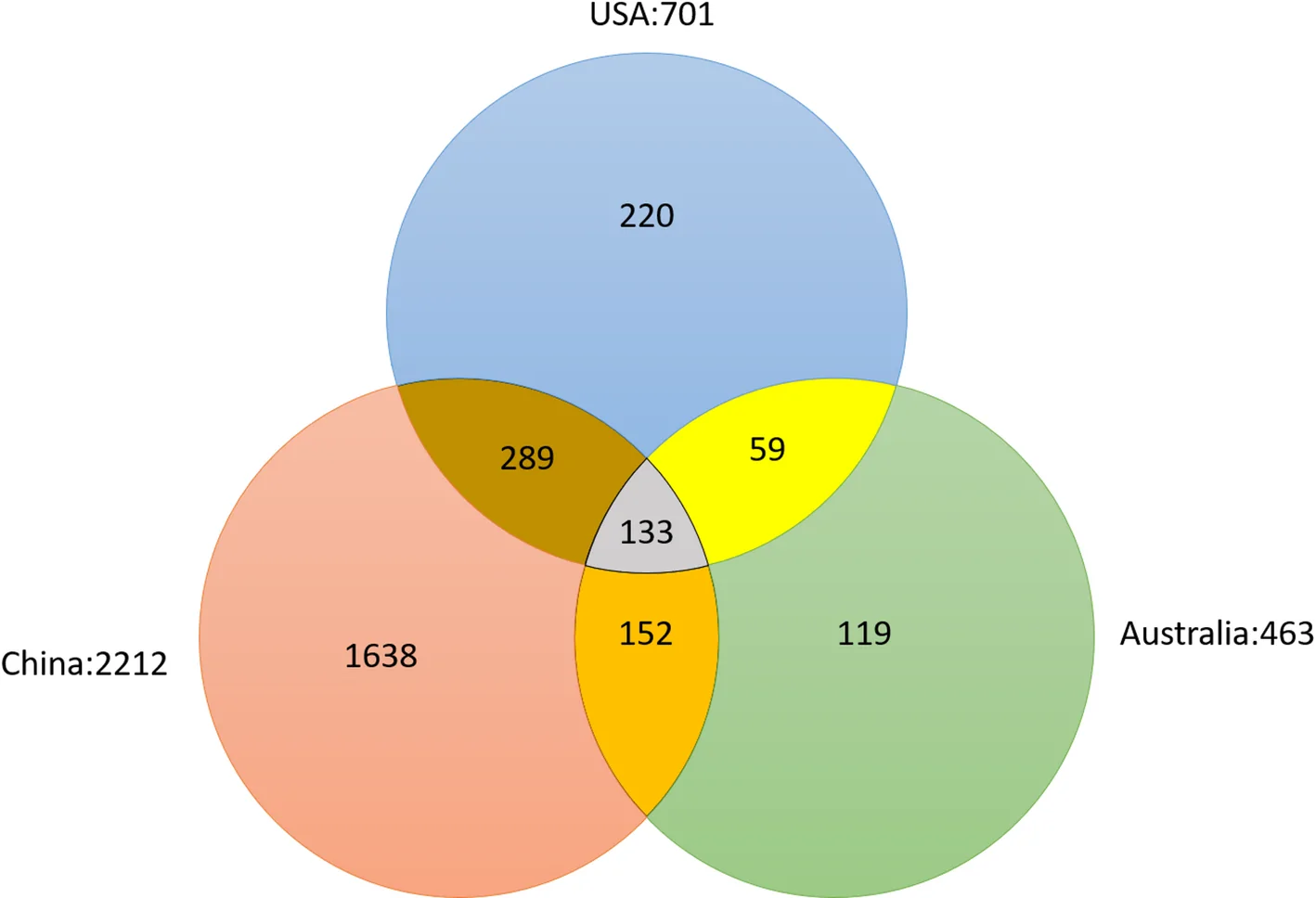
Venn diagram showing differentially expressed genes across three populations. China is represented in pink, the USA in blue, and Australia in green. Grey indicates DEGs common to all three populations

A total of 133 DEGs were shared across China, USA, and Australia. Additionally, 289 DEGs were common between China and the USA, 152 between China and Australia, and 59 between the USA and Australia. A Venn diagram (Fig. 2) illustrates DEG overlap. All 133 shared protein-coding DEGs were selected for further analysis.

###  Identification of hub genes via protein-protein interaction and gene–miRNA interaction

**Fig. 3:**
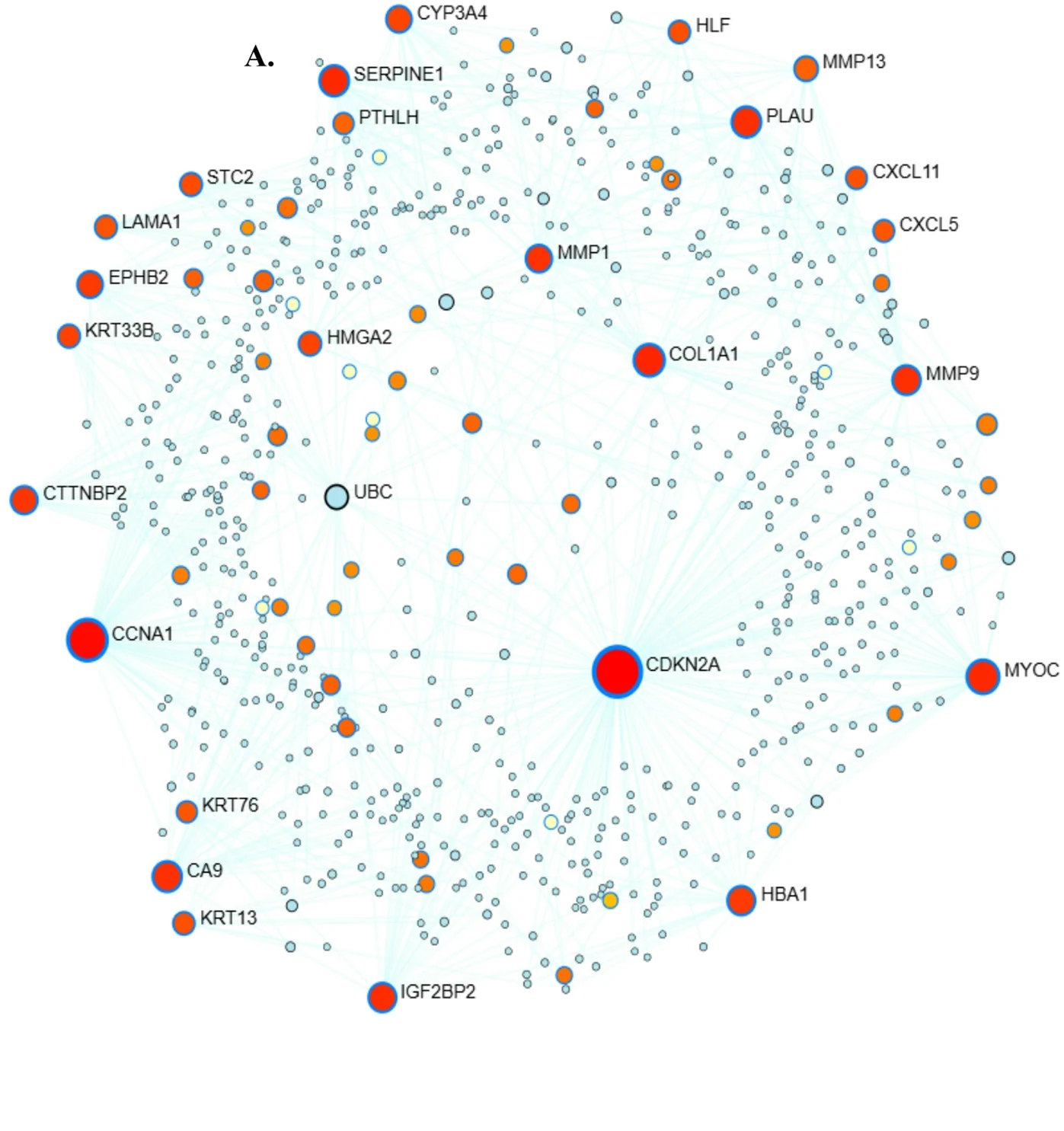
Protein–protein interaction (PPI) network of coding genes. Seed nodes are outlined in blue; their size and color intensity are proportional to the number of interactions. Other proteins are represented in cyan

**Fig. 4:**
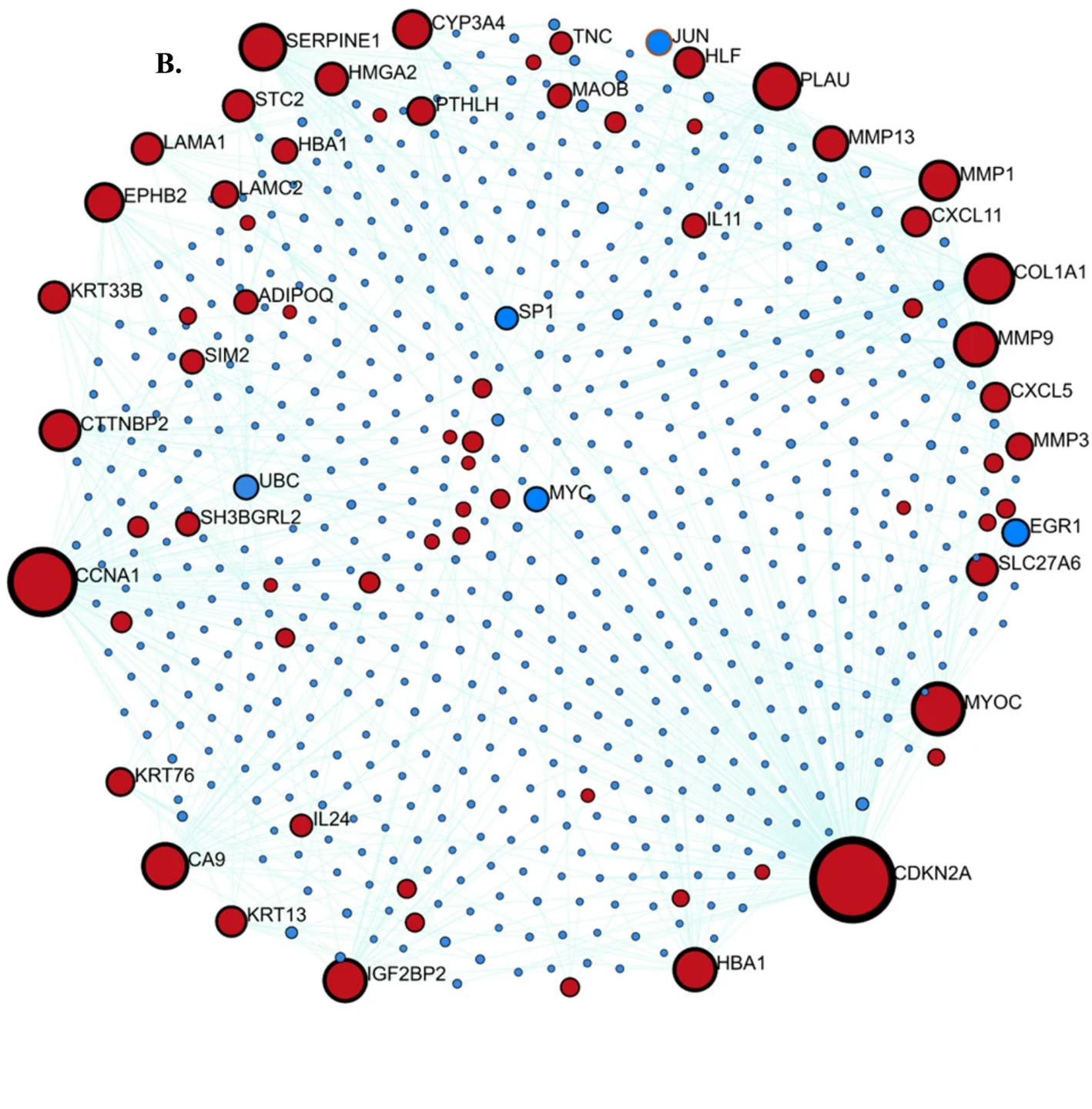
Gene–miRNA interaction network of coding genes. Seed nodes are shown in red with a black outline; their size and color intensity are proportional to the number of interactions. miRNAs are represented in dark blue

Of the 133 common DEGs, 104 were protein-coding, and 29 were non-coding. Non-coding genes were categorized based on their functional roles. A PPI network (Fig. 3) was built using 69 seed genes, resulting in 693 nodes and 870 edges, the PPI network constructed from protein coding DEGs showed a dense interaction pattern, with several highly connected nodes forming central clusters within the network. This indicates that a substantial proportion of the differentially expressed genes are functionally interconnected and may participate in shared biological pathways relevant to tongue cancer. Similarly, the gene–miRNA interaction network exhibited prominent hub nodes, suggesting coordinated post transcriptional regulation of key protein coding genes. The gene–miRNA interaction network (Fig. 4) was generated with 85 seed genes, producing 1145 nodes and 1599 edges, suggesting regulatory roles of miRNAs in DEG expression. Hub genes were identified from both networks for further analysis. To further highlight highly interactive hub gene subnetworks derived from the PPI and gene miRNA networks are shown in Supplementary Figure S1–S8.

###  Gene ontology (GO) and pathway enrichment analysis of protein coding genes

GO and KEGG analyses revealed that protein-coding DEGs were primarily associated with the extracellular matrix (ECM), particularly in disassembly and collagen catabolic processes critical for tissue remodeling and tumor invasion. Molecular function enrichment highlighted metallopeptidase and endopeptidase activities, indicating roles in ECM degradation and tumor microenvironment remodeling. Pathway enrichment showed overrepresentation in ECM–receptor interaction, IL-17 signaling, and human papillomavirus (HPV) infection pathways, which are linked to cellular adhesion, immune modulation, and metastasis. Disease enrichment analysis associated DEGs with chondrosarcoma, malignant neoplasm of the mouth, degenerative polyarthritis, keloid formation, endometriosis, colorectal carcinoma, bladder and stomach malignancies, periodontitis, and non-small cell lung carcinoma, underscoring shared molecular mechanisms in ECM remodeling, inflammation, and proliferation (Supplementary Tables 1–3) (Supplementary figure S9).

###  Selection of hub genes in tongue cancer

PPI network analysis identified hub genes, including MMP family members, COL family members, CDKN2A, CCNA1, SERPINE1, and CXCL5. Pathway analysis revealed strong interactions between MMP and COL genes, suggesting their coordinated role in tongue cancer progression (Supplementary Table 4).

###  Gene ontology and RNA–RNA Interaction analysis of non-coding RNAs

GO enrichment analysis of non-coding genes via RNA Enrich [24] (https://idrblab.cn/rnaenrich/) revealed associations with gland development and epithelial cell proliferation, suggesting regulatory roles in tissue morphogenesis and cellular dynamics. Reactome pathway analysis [25] indicated enrichment in IL-4 and IL-13 signaling, known for immune modulation, tissue remodeling, and inflammation. RNA–RNA interaction mapping identified eight lncRNAs, including TENM3-AS1, LINC00491, HMGA2-AS1, LINC02487, and DUXAP10, with significant interaction networks, suggesting roles in post-transcriptional regulation as molecular scaffolds or sponges (Supplementary Table 5) (Supplementary figures S1–S9). A list of all common coding and non-coding genes is provided in supplementary Tables 6–7.

##  Discussion

This systematic review provides an integrative analysis of the molecular landscape of tongue squamous cell carcinoma (TSCC), emphasizing its clinical burden and recent advances in biomarker discovery, diagnosis, and therapy. TSCC remains a major oral malignancy with high global morbidity and mortality, particularly in resource-limited regions.

###  Molecular and pathogenic complexity

Our findings highlight the multifactorial nature of TSCC, driven by molecular, cellular, and microenvironmental interactions. Coding DEGs were enriched in ECM disassembly, collagen catabolism, metallopeptidase activity, and pathways such as ECM–receptor interaction, IL-17, and HPV signaling, which are linked to invasiveness and immune modulation [26, 27]. Non-coding RNA analysis revealed enrichment in gland development, epithelial cell proliferation, and IL-4/IL-13 signaling [28]. RNA–RNA interaction profiling identified lncRNAs TENM3-AS1, LINC00491, HMGA2-AS1, LINC02487, and DUXAP10, potentially orchestrating post-transcriptional regulatory networks. Existing literature supports these findings, with ceRNA network analyses identifying oncogenic and tumor-suppressive lncRNAs such as SNHG6, KCNQ1OT1, and LINC00261 [29, 30]. Our study highlights TENM3-AS1, DUXAP10, and HMGA2-AS1 as novel TSCC-associated lncRNAs. Prior studies have reported deregulated lncRNAs in TSCC and OSCC—for example, Zhang et al. (2020) identified ~ 40 differentially expressed lncRNAs (including LINC00152, LINC01405, and RP11-54H7.4) [20] and constructed lncRNA–TF–mRNA networks. Similarly, DUXAP10 has been shown to drive proliferation in oral and esophageal SCC through p21 suppression [31], HMGA2-AS1 enhances growth and cisplatin resistance in esophageal SCC [32], and TENM3-AS1 was recently reported as a promoter of gastric cancer metastasis [33]. However, none of these three lncRNAs have been previously linked to TSCC, extending the ncRNA landscape in this malignancy. Mechanistically, lncRNAs such as H19, LINC00152, TENM3-AS1, and DUXAP10 act as molecular scaffolds, competing endogenous RNAs, and epigenetic regulators. H19 is a critical modulator of multiple cancer hallmarks [34, 35], while DUXAP10 promotes cell cycle progression and stem-like features [36]. Antisense RNAs, including TENM3-AS1, regulate gene expression through transcriptional interference, RNA masking, and chromatin remodeling, thereby shaping epithelial proliferation and differentiation [33]. Beyond their biomarker value, the therapeutic relevance of ncRNAs is increasingly evident: advances in RNA interference, antisense oligonucleotides, and nanoparticle-mediated delivery highlight their potential as direct targets in TSCC.

###  Advances in diagnostic and prognostic biomarkers

Unlike studies focusing on single lncRNA–miRNA–mRNA axes, our integrative approach combines GO, pathway, and RNA–RNA interaction analyses, enhancing biomarker discovery potential. Linking ECM remodeling and interleukin signaling with lncRNA interactions suggests an expanded set of candidate biomarkers for risk stratification and therapeutic targeting [37]. Recent work underscores the significance of MMPs (MMP1, MMP9, MMP10, MMP12, MMP13) and collagens (COL1A1, COL4A6, COL22A1) in shaping the TSCC microenvironment, driving invasion through ECM degradation and TNF signaling [38]. Tumor suppressor networks centered on CDKN2A interact with cell cycle regulators and MAPK signaling [39, 40], while inflammatory mediators such as IL-17, SERPINE1, and CXCL5 integrate immune signaling with ECM remodeling, reinforcing their biomarker potential [41].

###  Therapeutic strategies and outcomes

TSCC therapeutic strategies are evolving toward targeted and immunotherapy approaches. Molecular alterations in ECM dynamics and cytokine-mediated immune processes offer plausible therapeutic targets [27, 42]. Perturbing lncRNAs modulating ECM remodeling or immune signaling could sensitize tumors to anti-fibrotic or immunomodulatory agents [43]. Disease enrichment linking DEGs to related cancers and inflammatory disorders suggests common molecular vulnerabilities for therapeutic exploitation. Advances in lncRNA-targeted therapeutics, including nanoparticle-mediated RNA delivery, further highlight opportunities for clinical translation [44].

### Clinical validation and cellular origins of core signatures

**Fig. 5:**
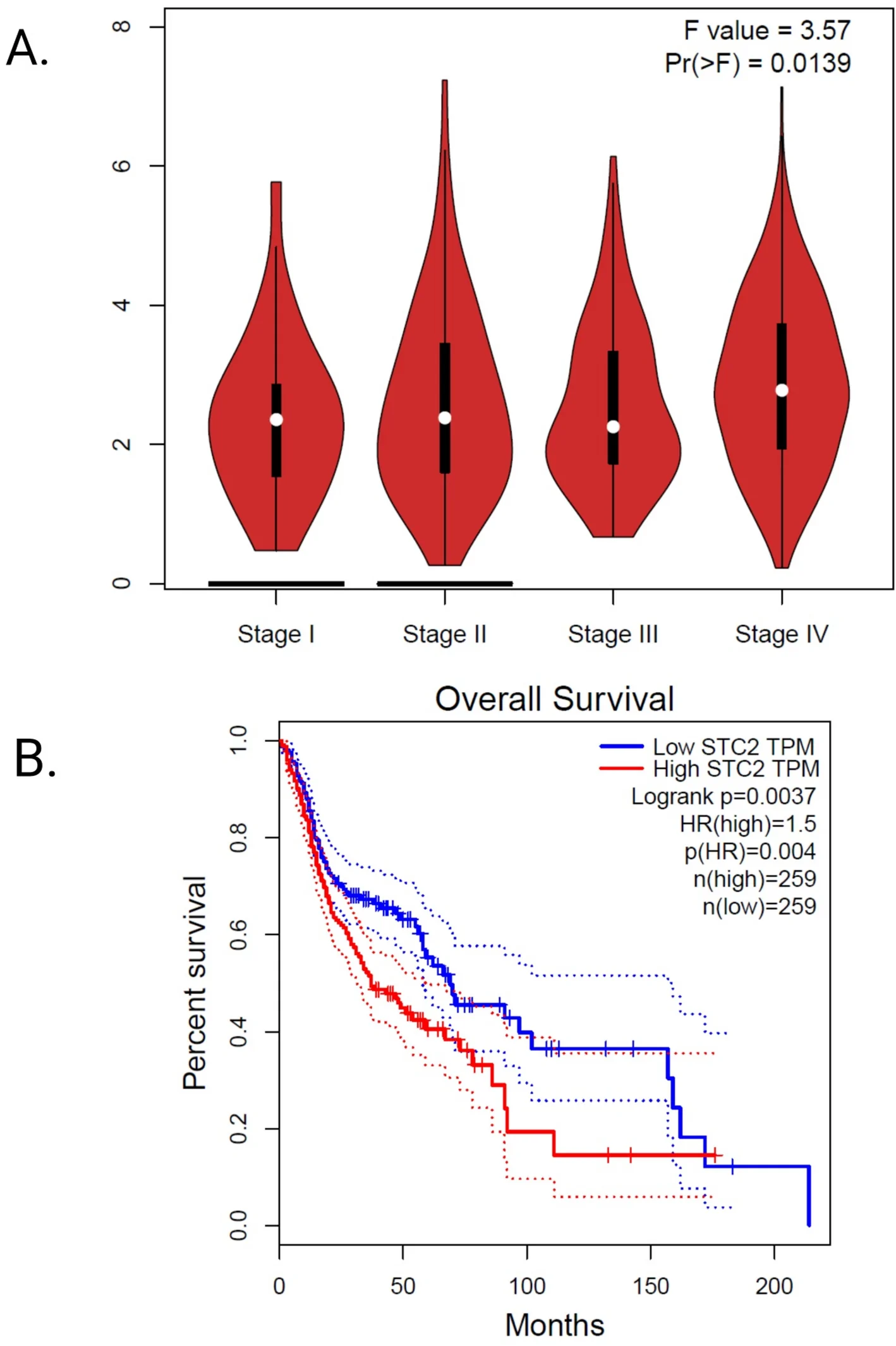
Clinical validation of *STC2* in the TCGA-HNSC cohort. **A** Violin plots showing significant correlation with tumor stage (*p* = 0.0139). **B** Kaplan–Meier analysis showing high *STC2* predicts poor overall survival (*p* = 0.0037)

To further contextualize our cross-population findings, we evaluated the clinical and cellular relevance of our core gene signature. Validation using the TCGA-HNSC cohort via the GEPIA [45] platform demonstrated that higher expression of key shared genes, including MMP3, STC2, and CXCL11, is associated with more advanced pathological stage and differential associations with overall survival (Fig. 5, supplementary figure S10-S11), supporting their potential prognostic relevance.

While our analytical framework prioritized cross-population robustness over direct single-cell integration, emerging scRNA-seq studies in head and neck squamous cell carcinoma (HNSCC) provide important biological context for these findings. MMP3, for example, has been identified as a marker of the partial epithelial–mesenchymal transition (pEMT) program in malignant cells and cancer-associated fibroblasts in oral cavity tumors, where it contributes to invasive behavior [46]. In parallel, CXCL11 expression is characteristic of an “immune-hot” tumor phenotype and localizes predominantly to inflammatory fibroblasts and macrophages [47], a feature with potential relevance for immunotherapy responsiveness. Notably, STC2 has recently been implicated in mediating tumor–vascular crosstalk in related squamous carcinomas [48], suggesting a conserved mechanism of hypoxic adaptation across HNSCC subsites.

Collectively, these observations suggest that our bulk-derived gene signature captures key multicellular programs spanning invasion, immune modulation, and hypoxic stress that contribute to tongue cancer progression.

###  Limitations of current research

Challenges include variability in sequencing and analysis methods, which can complicate cross-study comparability. All included datasets originated from studies conducted in China, the USA, or Australia, as these are currently the only regions with publicly available TSCC transcriptomic series in GEO. No comparable TSCC expression datasets from other regions, such as Europe, Asia, Africa, or Latin America, were identified, and our analysis therefore reflects the available data from these three populations. Technical variability arising from differences in sequencing platforms and chemistries (HiSeq vs. NovaSeq) was mitigated by performing differential expression analyses separately within each dataset using GEO2R (limma/DESeq2) and integrating results at the gene-signature level rather than merging raw expression matrices. This per-dataset normalization strategy reduces platform-specific biases during cross-population comparison, although residual batch effects cannot be fully excluded. In addition, the use of a stringent log_2_ fold change threshold may have excluded genes with moderate but biologically relevant expression differences; however, this conservative approach was chosen to improve the robustness and comparability of findings across heterogeneous public datasets. While recent multi-cohort oncology studies frequently employ high-dimensional algorithmic frameworks (such as LASSO Cox regression or deep machine-learning deconvolution) to generate prognostic risk scores, these models require immense, homogenous sample sizes to avoid overfitting. In this study, our strict quality assessment and risk of bias screening filtered out low-tier or site-heterogeneous series, resulting in a highly refined cumulative sample size (*n* = 68 total samples across the bulk-sequencing cohorts). Attempting to build complex machine-learning equations on a cohort of this size risks capturing platform-specific artifacts rather than genuine biological signals. Therefore, our primary, discovery-driven research design deliberately favors a conservative cross-population intersection approach. By filtering out population-specific “noise” through strict intersection and validating core hubs like STC2 and MMP3 against the massive independent TCGA-HNSC dataset (*n* = 518), we guarantee the biological generalizability of our 133-gene signature. Because further in silico modeling on limited public samples yields diminishing returns, the definitive validation of this core signature must happen at the bench using primary, patient-derived clinical samples (via qPCR or immunohistochemistry) within a localized trial framework.

###  Implications for clinical practice and future research

Integrating transcriptomic analyses GO, pathway, RNA–RNA interaction, and disease enrichment uncovers biologically and clinically relevant gene networks. Future research should validate candidate lncRNAs and their interaction partners in diverse TSCC cohorts. Developing noninvasive assays (e.g., circulating lncRNAs in saliva or plasma) targeting ECM related and immunomodulatory transcripts could improve early detection and personalized management. Hypothesis for multicultural integration: We propose that cross-population transcriptomic integration (China, USA, Australia) will identify conserved molecular signatures that transcend ethnic and environmental biases, defining robust biomarkers and therapeutic targets for TSCC. This approach enhances statistical power, reduces false associations, and prioritizes universally relevant pathways such as ECM remodeling, cytokine signaling, and ncRNA regulation. Validating these molecular networks across populations could advance precision medicine and help translate findings from research to clinical application.

###  Summary

**Fig. 6:**
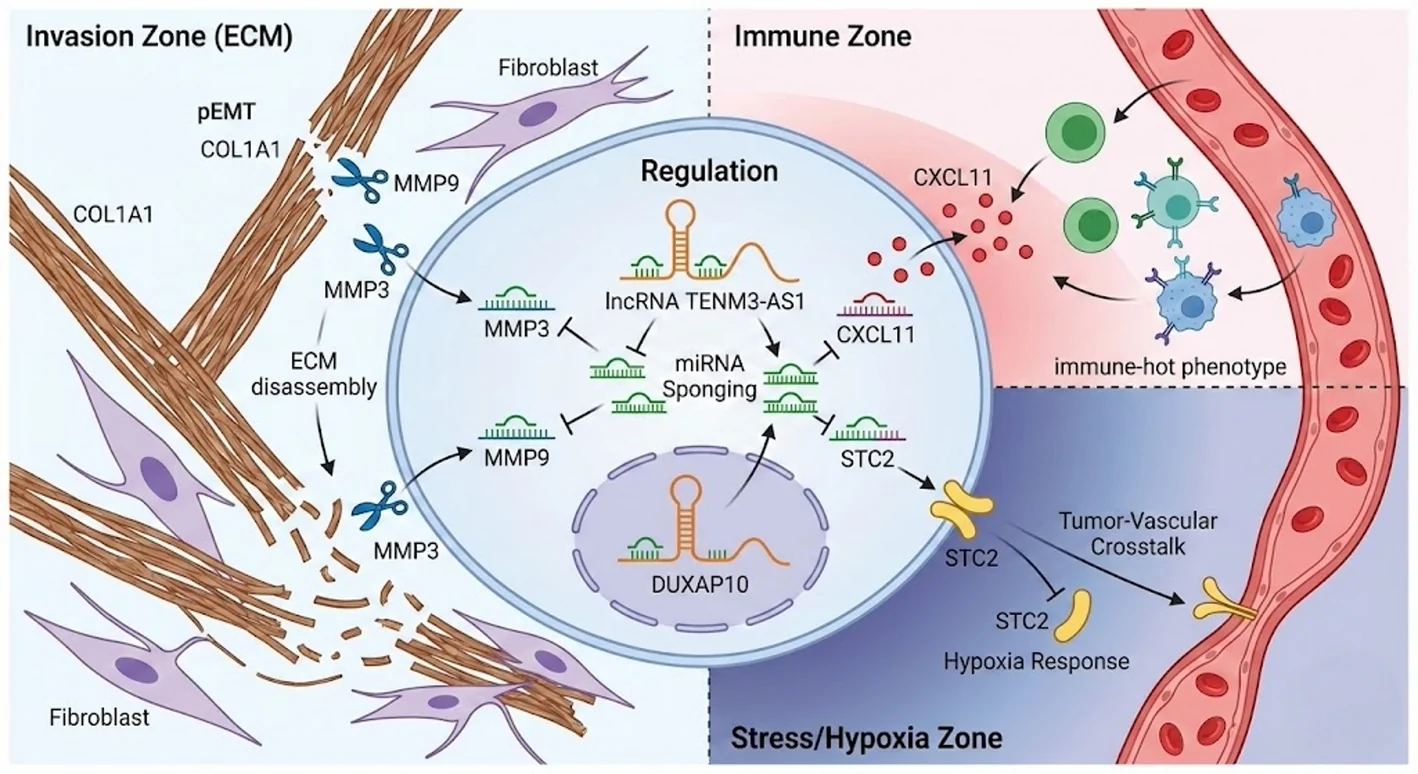
Schematic representation of the proposed tumor-microenvironment cross-talk zones (Invasion, Immune, Hypoxia) driven by the identified gene signature

In summary, our integrative analysis overcomes the limitations of single-cohort studies by defining a robust, cross-population transcriptomic signature for TSCC derived from diverse patient groups in China, the USA, and Australia. Beyond identifying common DEGs, we mapped these genes to a coordinated tumor-microenvironment cross-talk mechanism (Fig. 6). This model highlights three critical pathogenic zones: [1] an invasive front driven by ECM-remodeling enzymes (*MMP3*,* MMP9*,* COL1A1*); [2] an immune-modulatory zone characterized by *CXCL11* signaling; and [3] a stress-response niche marked by *STC2* upregulation. Furthermore, we uncovered a non-coding regulatory layer, identifying lncRNAs such as *TENM3-AS1* and *DUXAP10* as potential upstream “sponges” that orchestrate these downstream effectors.

##  Conclusion

This systematic review elucidates the critical molecular mechanisms underlying tongue cancer, moving beyond simple gene lists to identify functional networks that transcend ethnic variability. We identified a core signature of 133 genes, with *MMP* and *COL* family members, *CDKN2A*, *SERPINE1*, and *CXCL11* emerging as central regulators of invasion and immune surveillance. Crucially, we bridged the gap between bioinformatic discovery and clinical utility by validating these targets in the independent TCGA-HNSC cohort, demonstrating that high expression of *STC2* and *MMP3* is significantly associated with advanced tumor stage and poor overall survival (Fig. 5, Supplementary Fig. 10–11). These findings provide a biologically validated foundation for developing precision biomarkers and ncRNA-targeted therapies for tongue cancer management.

## Supplementary Information


Supplementary Material 1.



Supplementary Material 2.


## Data Availability

No datasets were generated or analysed during the current study.

## Materials availability

GEO Accession number along with their links.

**Table:**

Accession numbers	Links
GSE143950	https://www.ncbi.nlm.nih.gov/geo/query/acc.cgi?acc=GSE143950
GSE184616	https://www.ncbi.nlm.nih.gov/geo/query/acc.cgi?acc=GSE184616
GSE149008	https://www.ncbi.nlm.nih.gov/geo/query/acc.cgi?acc=GSE149008
GSE156178	https://www.ncbi.nlm.nih.gov/geo/query/acc.cgi?acc=GSE156178
GSE131568	https://www.ncbi.nlm.nih.gov/geo/query/acc.cgi?acc=GSE131568

**Table:**

Databases used	Links
Enrich	https://maayanlab.cloud/Enrichr
KEGG	https://www.genome.jp/kegg/pathway.html
NetworkAnalyst PPI	https://www.networkanalyst.ca/NetworkAnalyst/Secure/vis/NetworkView.xhtml
NetworkAnalyst Gene mi-RNA	https://www.networkanalyst.ca/NetworkAnalyst/Secure/vis/NetworkView.xhtml
