# Supplementary material for: Integrative transcriptomic analysis identifies shared coding and non-coding biomarkers in tongue squamous cell carcinoma across diverse populations
Source: Discov Oncol. 2026 Aug 27;17:1253. doi: 10.1007/s12672-026-05428-9 (PMC13522316; doi:10.1007/s12672-026-05428-9)
Supplement: Supplementary file 1 [file 12672_2026_5428_MOESM1_ESM.docx]

| **S.No.** | **GO:BP** | **P-value** | **Associated Genes** |
| --- | --- | --- | --- |
| 1 | External Encapsulating Structure Organization (GO:0045229) | 0.00 | MMP12, MMP11, ADAMTS2, MMP13, MMP1, COL22A1, MMP3, COL4A6, TGFBI, ADAMTS12, MMP9, MMP10 |
| 2 | Extracellular Structure Organization (GO:0043062) | 0.00 | MMP12, MMP11, ADAMTS2, MMP13, MMP1, COL22A1, MMP3, COL4A6, TGFBI, ADAMTS12, MMP9, MMP10 |
| 3 | Extracellular Matrix Organization (GO:0030198) | 0.00 | MMP1, COL22A1, COL12A1, MMP3, ADAMTS12, MMP9, MMP10, COL1A1, MMP12, MMP11, ADAMTS2, MMP13, COL4A6, TGFBI |
| 4 | Collagen Catabolic Process (GO:0030574) | 0.00 | MMP12, MMP11, MMP13, MMP1, MMP3, MMP9, MMP10 |
| 5 | Extracellular Matrix Disassembly (GO:0022617) | 0.00 | MMP12, MMP11, MMP13, MMP1, MMP3, MMP9, MMP10 |
| 6 | Cellular Component Disassembly (GO:0022411) | 0.00 | MMP12, MMP11, MMP13, MMP1, MMP3, MMP9, MMP10 |
| 7 | Cellular Response to UV-A (GO:0071492) | 0.00 | MMP12, MMP1, MMP3, MMP9 |
| 8 | Response to UV-A (GO:0070141) | 0.00 | MMP12, MMP1, MMP3, MMP9 |
| 9 | Negative Regulation of Cell-Matrix Adhesion (GO:0001953) | 0.00 | MMP12, MYOC, CDKN2A, SERPINE1 |
| 10 | Endodermal Cell Differentiation (GO:0035987) | 0.00 | COL12A1, HMGA2, INHBA, MMP9 |

Table 1: Gene ontology of biological processes top 10

| **S.No.** | **GO:MF** | **P-value** | **Associated Genes** |
| --- | --- | --- | --- |
| 1 | Metalloendopeptidase Activity (GO:0004222) | 0.00 | MMP12, MMP11, ADAMTS2, MMP13, MMP1, ADAM12, MMP3, ADAMTS12, MMP9, MMP10 |
| 2 | Metallopeptidase Activity (GO:0008237) | 0.00 | MMP12, MMP11, ADAMTS2, MMP13, MMP1, ADAM12, MMP3, ADAMTS12, MMP9, MMP10 |
| 3 | Serine-Type Endopeptidase Activity (GO:0004252) | 0.00 | MMP12, MMP11, MMP13, PLAU, MMP1, MMP3, MMP9, MMP10 |
| 4 | Endopeptidase Activity (GO:0004175) | 0.00 | MMP12, MMP11, ADAMTS2, MMP13, PLAU, MMP1, ADAM12, MMP3, ADAMTS12, MMP9, MMP10 |
| 5 | Serine-Type Peptidase Activity (GO:0008236) | 0.00 | MMP12, MMP11, MMP13, PLAU, MMP1, MMP3, MMP9, MMP10 |
| 6 | Receptor Ligand Activity (GO:0048018) | 0.00 | NPPC, IL11, ARTN, ADIPOQ, IL24, FAM3B, FAM3D, INHBA, PTHLH, APLN |
| 7 | Cytokine Activity (GO:0005125) | 0.00 | IL11, CXCL11, IL24, FAM3B, FAM3D, INHBA, CXCL5 |
| 8 | Hormone Activity (GO:0005179) | 0.00 | NPPC, ADIPOQ, INHBA, PTHLH, APLN |
| 9 | Calcium Ion Binding (GO:0005509) | 0.00 | S100A7A, MMP12, NELL2, SCIN, MMP13, SYT12, MMP1 |
| 10 | CXCR Chemokine Receptor Binding (GO:0045236) | 0.00 | CXCL11, CXCL5 |

Table 2: Gene ontology of molecular functions top 10

| **S.No.** | **GO:CC** | **P-value** | **Associated Genes** |
| --- | --- | --- | --- |
| 1 | Collagen-Containing Extracellular Matrix (GO:0062023) | 0.00 | MYOC, LAMA1, COL22A1, COL12A1, SERPINE1, ADIPOQ, LAMC2, MMP9, COL1A1, ESM1, MFAP2, COL4A6, AMTN, TGFBI, CTHRC1 |
| 2 | Basement Membrane (GO:0005604) | 0.00 | LAMA1, COL22A1, AMTN, COL4A6 |
| 3 | Serine Protease Inhibitor Complex (GO:0097180) | 0.00 | PLAU, SERPINE1 |
| 4 | Keratin Filament (GO:0045095) | 0.00 | KRT13, KRT76, KRT84 |
| 5 | Intracellular Organelle Lumen (GO:0070013) | 0.00 | COL1A1, MMP11, CDKN2A, STC2, COL22A1, COL12A1, COL4A6, AMTN, HMGCS2, HBA1, MMP9, MUC21 |
| 6 | Endoplasmic Reticulum Lumen (GO:0005788) | 0.00 | COL1A1, STC2, COL22A1, COL12A1, COL4A6, AMTN |
| 7 | Intermediate Filament (GO:0005882) | 0.01 | KRT13, KRT76, KRT84 |
| 8 | Cell Projection Membrane (GO:0031253) | 0.02 | PDPN, ATP6V0A4, CNGB1 |
| 9 | Intracellular Cyclic Nucleotide Activated Cation Channel Complex (GO:0017071) | 0.03 | CNGB1 |
| 10 | Inhibitory Synapse (GO:0060077) | 0.04 | SYT12 |

Table 3: Gene ontology of cellular components top 10

| **S.No.** | **Pathway(KEGG)** | **P-value** | **Associated Genes** |
| --- | --- | --- | --- |
| 1 | ECM-receptor interaction | 0.00 | COL1A1, LAMA1, LAMB4, TNC, COL4A6, LAMC2 |
| 2 | IL-17 signaling pathway | 0.00 | S100A7A, MMP13, MMP1, MMP3, MMP9, CXCL5 |
| 3 | PPAR signaling pathway | 0.00 | MMP1, ADIPOQ, SLC27A6, HMGCS2, PLIN1 |
| 4 | Human papillomavirus infection | 0.00 | COL1A1, CCNA1, LAMA1, LAMB4, TNC, COL4A6, ATP6V0A4, LAMC2, OASL |
| 5 | Rheumatoid arthritis | 0.00 | IL11, MMP1, MMP3, ATP6V0A4, CXCL5 |
| 6 | Amoebiasis | 0.00 | COL1A1, LAMA1, LAMB4, COL4A6, LAMC2 |
| 7 | Relaxin signaling pathway | 0.00 | COL1A1, MMP13, MMP1, COL4A6, MMP9 |
| 8 | Focal adhesion | 0.00 | COL1A1, LAMA1, LAMB4, TNC, COL4A6, LAMC2 |
| 9 | Bladder cancer | 0.00 | CDKN2A, MMP1, MMP9 |
| 10 | Small cell lung cancer | 0.00 | LAMA1, LAMB4, COL4A6, LAMC2 |

Table 4: Pathway analysis of DEGs, top 10

| **DO(DisGeNet)** | **P-value** | **Associated Genes** |
| --- | --- | --- |
| Chondrosarcoma | 0.00 | IL11, MMP1, CDKN2A, SERPINE1, ADIPOQ, MMP3, TNC, HMGA2, INHBA, MMP9, PTHLH, MMP12, ADAMTS2, MMP13, PLAU, PDPN, EPHB2 |
| Malignant neoplasm of mouth | 0.00 | MMP1, CDKN2A, SERPINE1, TNC, KRT76, HOXD11, MMP9, HOXD10, PTHLH, MMP11, ESM1, PLAU, PDPN, CA9, HMGCS2, EPHB2, CTHRC1 |
| Degenerative polyarthritis | 0.00 | IL11, MMP1, CDKN2A, SERPINE1, ADIPOQ, MMP3, TNC, HMGA2, INHBA, ADAMTS12, MMP9, MMP10, PTHLH, APLN, COL1A1, MMP12, MMP13, FAP, PLAU, ADAM12, TGFBI, EPHB2, CNGB1 |
| Keloid | 0.00 | COL1A1, MMP13, MMP1, SERPINE1, ADAM12, MMP3, INHBA, TGFBI, CYP3A4, MMP9, CTHRC1 |
| Endometriosis | 0.00 | IL11, MAOB, MMP1, CDKN2A, COL12A1, SERPINE1, ADIPOQ, MMP3, TNC, HMGA2, INHBA, MMP9, CXCL5, COL1A1, MMP12, MMP13, PLAU, STC2, TGFBI, EPHB2 |
| Colorectal Carcinoma | 0.00 | ONECUT2, LAMA1, COL12A1, SERPINE1, IL24, TNC, LAMC2, CYP3A4, HOXD10, CXCL5, PTHLH, FUT6, CYP27B1, ESM1, CST1, PLAU, STC2, CA9, HMGCS2, IGF2BP2, EPHB2, CSMD2, NKX2-3, STRA6, CTHRC1, IL11, MMP1, CDKN2A, ADIPOQ, MMP3, HMGA2, MMP9, MMP12, CCNA1, MMP11, MMP13, AADAC, FAP, TGFBI, CNGB1 |
| Malignant neoplasm of urinary bladder | 0.00 | MMP1, CDKN2A, SERPINE1, IL24, MMP3, HMGA2, SOX11, LAMC2, CYP3A4, MMP9, CXCL5, MMP10, MMP12, NELL2, CCNA1, MMP11, ESM1, MMP13, AADAC, PLAU, ADAM12, PDPN, CA9, SLC38A3 |
| Periodontitis | 0.00 | MMP12, IL11, MMP13, MMP1, CDKN2A, SERPINE1, MMP3, TNC, EPHB2, MMP9, CXCL5, JCHAIN |
| Non-Small Cell Lung Carcinoma | 0.00 | MAOB, SERPINE1, IL24, TNC, LAMC2, CYP3A4, CXCL5, PTHLH, S100A7A, CYP27B1, ESM1, CST1, PLAU, PDPN, CA9, EPHB2, CTHRC1, IL11, MMP1, CDKN2A, ADIPOQ, MMP3, HMGA2, INHBA, MMP9, APLN, MMP12, MMP11, CXCL11, FAP, ARTN, ADAM12, TGFBI, SIM2 |
| Malignant neoplasm of stomach | 0.00 | SERPINE1, IL24, LAMC2, HOXD10, CXCL5, FUT6, ESM1, CST1, SCIN, PLAU, STC2, PDPN, FAM3B, CA9, EPHB2, CTHRC1, IL11, MMP1, CDKN2A, ADIPOQ, MMP3, HMGA2, SOX11, INHBA, MMP9, MMP10, SPOCD1, COL1A1, MMP12, CCNA1, MMP11, MMP13, FAP, ADAM12, TGFBI |

Table 5: Disease ontology of DEGs, top10

| Coding Genes | | | | | | | |
| --- | --- | --- | --- | --- | --- | --- | --- |
| AADAC | CNGB1 | CYP3A4 | HMGA2 | KRT76 | MMP13 | PLAU | SLC27A6 |
| ADAM12 | COL12A1 | EN1 | HMGCS2 | KRT84 | MMP3 | PLIN1 | SLC38A3 |
| ADAMTS12 | COL1A1 | EPHB2 | HOXD10 | LAMA1 | MMP9 | PPP4R4 | SLITRK5 |
| ADAMTS2 | COL22A1 | ESM1 | HOXD11 | LAMB4 | MUC21 | PTHLH | SOX11 |
| ADIPOQ | COL4A6 | FAM3B | HTR7 | LAMC2 | MYO3B | PTPRH | SPOCD1 |
| AMTN | CREG2 | FAM3D | IGF2BP2 | LIPG | MYOC | RPTN | STC2 |
| APLN | CSMD2 | FAP | IGHA2 | LRFN2 | NELL2 | S100A7A | STEAP1B |
| ARTN | CST1 | FUT6 | IL11 | MAOB | NKX2-3 | SAMD5 | STRA6 |
| ATP6V0A4 | CTHRC1 | GPR158 | IL24 | MFAP2 | NPPC | SCIN | SYT12 |
| CA9 | CTTNBP2 | GYS2 | INHBA | MMP1 | OASL | SERPINE1 | TGFBI |
| CCNA1 | CXCL11 | HBA1 | JCHAIN | MMP10 | ONECUT2 | SH2D5 | TNC |
| CDKN2A | CXCL5 | HBA2 | KRT13 | MMP11 | PAX1 | SH3BGRL2 | ZNF114 |
| CFAP251 | CYP27B1 | HLF | KRT33B | MMP12 | PDPN | SIM2 | ZNF469 |

Table 6: List of all common coding genes found across 3 population

| Non-coding genes | | | | |
| --- | --- | --- | --- | --- |
| ANKRD20A5P | HMGA2-AS1 | LINC02910 | LOC105374764 | SLC12A5-AS1 |
| C10ORF55 | KLHDC7B-DT | LCAL1 | LOC105375242 | TENM3-AS1 |
| CYP4F29P | LINC00491 | LOC101929532 | LOC105376081 | TMPRSS11BNL |
| DUXAP10 | LINC01395 | LOC105370455 | LOC105376806 | WTAPP1 |
| FAM3D-AS1 | LINC01697 | LOC105371446 | LOC110091776 | ZFPM2-AS1 |
| FTLP10 | LINC02487 | LOC105372641 | MIR3606 | - |

Table 7: List of all common non-coding genes found across 3 population
