# Supplementary material for: Integrative transcriptomic analysis identifies shared coding and non-coding biomarkers in tongue squamous cell carcinoma across diverse populations
Source: Discov Oncol. 2026 Aug 27;17:1253. doi: 10.1007/s12672-026-05428-9 (PMC13522316; doi:10.1007/s12672-026-05428-9)
Supplement: Supplementary file 2 [file 12672_2026_5428_MOESM2_ESM.docx]

**Supplementary Figure S1**. Interaction network of the long non-coding RNA (lncRNA) TENM3-AS1. The red circle represents the query gene (TENM3-AS1), blue boxes indicate interacting miRNAs, and white boxes denote associated coding genes. The network illustrates potential regulatory relationships between TENM3-AS1, miRNAs, and their target genes.

**Supplementary Figure S2**. Interaction network of the long non-coding RNA (lncRNA) LINC00491. The red circle represents the query gene (LINC00491), blue boxes indicate interacting miRNAs, and white boxes denote associated coding genes. The network illustrates potential regulatory relationships between LINC00491, miRNAs, and their target genes.

**Supplementary Figure S3.** Interaction network of the long non-coding RNA (lncRNA) HMGA2-AS1. The red circle represents the query gene (HMGA2-AS1), white boxes denote associated coding genes. The network illustrates potential regulatory relationships between HMGA2-AS1, and their target genes.

**Supplementary Figure S4**  Interaction network of the long non-coding RNA (lncRNA) LINC02487. The red circle represents the query gene (LINC02487), white boxes denote associated coding genes. The network illustrates potential regulatory relationships between LINC02487, and their target genes.

**Supplementary Figure S5** Interaction network of the long non-coding RNA (lncRNA) DUXAP10. The red circle represents the query gene (DUXAP10), white boxes denote associated coding genes. The network illustrates potential regulatory relationships between DUXAP10, and their target genes.

**Supplementary Figure S6.** Interaction network of the long non-coding RNA (lncRNA) ZFPM2-AS1. The red circle represents the query gene (ZFPM2-AS1), blue boxes indicate interacting miRNAs, and white boxes denote associated coding genes. The network illustrates potential regulatory relationships between ZFPM2-AS1, miRNAs, and their target genes.

**Supplementary Figure S7.** Extracted protein–protein interaction (PPI) subnetwork showing highly interactive genes. Nodes representing genes with the highest number of interactions are colored dark blue, with node size proportional to the interaction degree (number of connections). This subnetwork was derived from the full PPI network in Figure 2A.

**Supplementary Figure S8.** Gene–miRNA interaction network extracted from Figure 2B. Query genes are represented by red-orange circles, with their color intensity and size proportional to the number of interactions. miRNAs are represented by dark blue squares.

**Supplementary Figure S9.** Gene Ontology (GO) enrichment analysis of non-coding RNA genes. The top enriched GO terms are shown for non-coding genes based on their associated biological processes, molecular functions, and cellular components. Bar length represents the number of genes mapped to each term, GO enrichment was performed using RNA enrich.

**Supplementary Figure S10.** CXCL11 expression across tumor stage and its association with overall survival. **(A)** Violin plots showing CXCL11 expression (TPM) across tumor stages I–IV. White dots indicate median expression; black bars represent the interquartile range. Differences across stages were assessed by one-way ANOVA (F = 4.65, p = 0.00324).

**(B)** Kaplan–Meier analysis of overall survival stratified by high versus low CXCL11 expression. Survival curves were compared using the log-rank test (log-rank p = 0.28); hazard ratio (high vs. low) = 0.86. Dotted lines indicate 95% confidence intervals.

**Supplementary Figure S11.** MMP3 expression across tumor stage and association with overall survival. **(A)** Violin plots showing MMP3 expression (TPM) across tumor stages I–IV. White dots indicate median expression; black bars represent the interquartile range. Differences among stages were evaluated by one-way ANOVA (F = 3.14, *p* = 0.025).
**(B)** Kaplan–Meier analysis of overall survival stratified by high versus low MMP3 expression. Survival differences were assessed using the log-rank test (log-rank *p* = 0.23); hazard ratio (high vs. low) = 1.2. Dotted lines indicate 95% confidence intervals.
